# Mesoporous PdBi nanocages for enhanced electrocatalytic performances by all-direction accessibility and steric site activation†

**DOI:** 10.1039/d1sc06314f

**Published:** 2022-02-28

**Authors:** Dawei Du, Qinghong Geng, Lian Ma, Siyu Ren, Jun-Xuan Li, Weikang Dong, Qingfeng Hua, Longlong Fan, Ruiwen Shao, Xiaoming Wang, Cuiling Li, Yusuke Yamauchi

**Affiliations:** a Key Laboratory of Cluster Science, Ministry of Education, Beijing Key Laboratory of Photoelectronic/Electrophotonic Conversion Materials, School of Chemistry and Chemical Engineering, Beijing Institute of Technology Beijing 100081 China licuiling@bit.edu.cn; b Beijing Advanced Innovation Center for Intelligent Robots and Systems and Institute of Engineering Medicine, Beijing Institute of Technology Beijing 100081 China; c Key Laboratory for Preparation and Application of Ordered Structural Materials of Guangdong Province, Department of Chemistry, Shantou University Shantou 515063 China; d International Center for Materials Nanoarchitectonics (MANA), National Institute for Materials Science (NIMS) Tsukuba 305-0044 Japan y.yamauchi@uq.edu.au; e School of Chemical Engineering and Australian Institute for Bioengineering and Nanotechnology (AIBN), The University of Queensland Brisbane 4072 Australia

## Abstract

An effective yet simple approach was developed to synthesize mesoporous PdBi nanocages for electrochemical applications. This technique relies on the subtle utilization of the hydrolysis of a metal salt to generate precipitate cores *in situ* as templates for navigating the growth of mesoporous shells with the assistance of polymeric micelles. The mesoporous PdBi nanocages are then obtained by excavating vulnerable cores and regulating the crystals of mesoporous metallic skeletons. The resultant mesoporous PdBi nanocages exhibited excellent electrocatalytic performance toward the ethanol oxidation reaction with a mass activity of 3.56 A mg^−1^_Pd, specific activity of 17.82 mA cm^−2^ and faradaic efficiency of up to 55.69% for C1 products.

## Abstract 2

An effective yet simple approach was developed to synthesize mesoporous PdBi nanocages for electrochemical applications.

## Graphical abstract

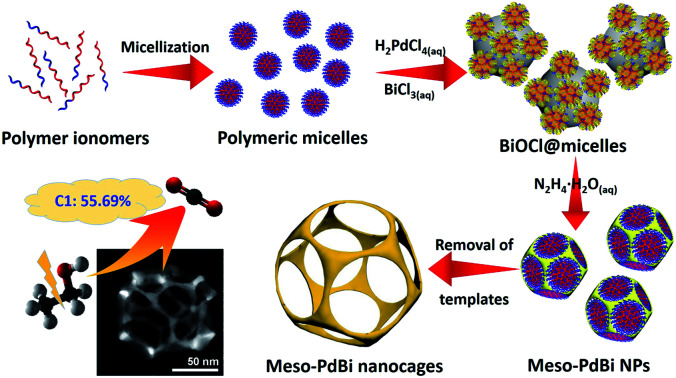

## Introduction

Nanoarchitectured materials with open surface features, such as porous structures,^1,2^ nanocages,^3–5^ nanoframes,^6–8^ and nanocrystals with high-index facets,^9,10^ have been widely investigated as a means of optimizing the efficiency of electrocatalysts. In particular, nanocages consisting of joint nanoridges can boost superior catalytic performance due to their three-dimensional accessibility and less susceptible structures. For example, an extraordinary mass activity of 0.75 A mg^−1^_Pt for the oxygen reduction reaction was achieved by unique Pt octahedral nanocages with a wall thickness of several atomic layers transformed from conformal Pd@Pt octahedra by interior erosion.^3^ Thus, creating metallic nanoarchitectures with a high number of accessible active surface sites to boost activity and durability is likely to advance the commercialization of fuel cells, which directly convert the chemical energy stored in chemicals into electrical energy.^11–15^

New approaches which provide better control of pore sizes and pore arrangement by precisely tailoring the micelle assembly process have been developed for the synthesis of mesoporous metals with improved electrocatalytic properties.^1,2,16–19^ However, it is difficult to balance the surface area and accessibility of the active sites as small pore sizes confer a large surface area but limited accessibility, and large pore sizes with favorable accessibility usually sacrifice the surface area.^20–22^ The new family of mesoporous nanocages reported here offers a new paradigm for ingeniously solving this contradiction by enhancing the accessibility of the generated active sites from all directions, rather than just from the outer surfaces. Nanocages have been demonstrated in Pt-based materials by selectively removing less stable compositions or evolving phase segregation of different components.^23,24^ However, mesoporous nanocages have not been reported probably due to the challenge of regulating micelle assembly on heterogeneous surfaces during the porous metal growth process.

**Fig. 1:**
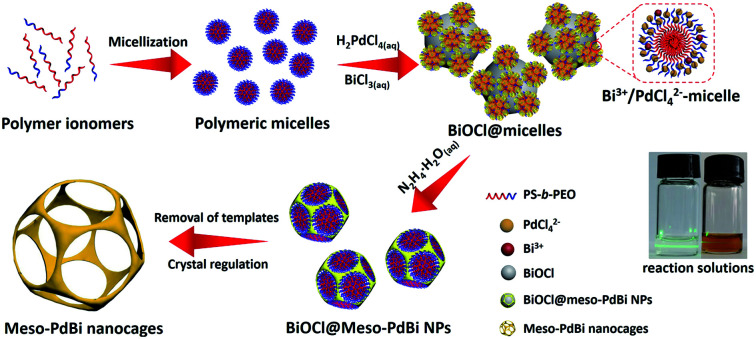
Schematic illustration of the preparation of meso-PdBi nanocages.

Here, we present a simple yet general method for synthesizing mesoporous PdBi nanocages with skeletons full of concave surfaces by an all-wet synthetic approach, which uses flexible polymeric micelles derived from polystyrene-*b*-poly(ethylene oxide) (PS-*b*-PEO) as soft-templates to guide the growth of mesoporous metals (Fig. 1).

## Results and discussion

**Fig. 2:**
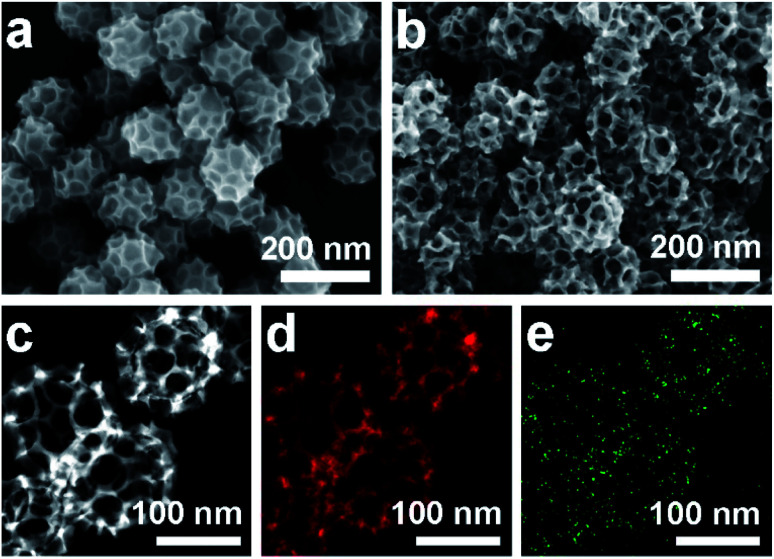
(a and b) SEM images of the obtained products (a) before and (b) after HCl etching treatment. (c–e) HAADF-STEM image and corresponding elemental maps (Pd (red) and Bi (green)).

Scanning electron microscope (SEM) and transmission electron microscope (TEM) images clearly show solid cores surrounded by a thin layer of mesoporous shells (Fig. 2a and S1†). Based on the elemental maps and cross-sectional compositional line profiles of an individual mesoporous nanoparticle (Fig. S2†), elemental segregation is distinctly observed. The Bi, Cl and O elements are enriched in the central region, whereas Pd is distributed all over the particle. To reveal the exact composition of the core, initial white precipitates formed after mixing the solutions were collected and characterized by wide-angle X-ray diffraction (XRD). All the diffraction peaks could be well indexed to the diffraction patterns of BiOCl (JCPDS card no. 73-2060) (Fig. S3†). These results confirm the formation of a mesoporous shell with a solid BiOCl core. In this synthetic procedure, once the acidic BiCl_3_ solution is added to the less acidic polymeric micelle solution, uniform BiOCl nanoparticles are formed in the solution due to the hydrolysis of BiCl_3_ after the rapid pH increase (Fig. S4a†). The remaining Bi^3+^ and [PdCl_4_]^2−^ ions are selectively incorporated into the hydrophilic shells of polymeric micelles because of the interactions between metal ions and the hydrophilic chains. When the reducing agent (hydrazine) is added, metallic crystals grow with the confinement effect of PEO blocks. Meanwhile, the hydrophobic cores without metal ions form the mesopores. As a result, the BiOCl nanoparticles then serve as hard-templates for the growth of mesoporous shells with the assistance of PS-*b*-PEO micelles. Mesoporous shells develop within 15 min due to the strong reducing capability of hydrazine (Fig. S4†).

After removal of the sacrificial BiOCl cores, mesoporous nanocages with highly open structures were obtained as shown in SEM and TEM images (Fig. 2b and S5†). No difference in particle size and pore structure is observed, indicating that the porous constructions do not collapse after removing the cores. The particle size and pore size of the obtained mesoporous nanocages are statistically 125 ± 3.0 nm and 26.7 ± 0.8 nm, respectively (Fig. S5†). The high-angle annular dark-field scanning transmission electron microscope (HAADF-STEM) image (Fig. 2c and S5†) reveals that the pore wall skeletons (thickness of 7.6 ± 0.3 nm) are interconnected together at the external surfaces, demonstrating the successful formation of mesoporous nanocages with hollow interiors. Interestingly, the interior size and overall particle size can be tuned by controlling the hydrolysis of Bi^3+^ by adjusting the acid concentration in the reaction condition. A higher acid concentration results in less BiOCl cores with a larger size, which is consistent with the pH-dependence of Bi^3+^ hydrolysis (Fig. S6†).^25,26^ Moreover, the acid concentration imparts pore regulations to the mesoporous shells. A relatively low growth speed at a higher acid concentration benefits the formation of ordered mesoporous shells, thus resulting in well-organized nanocages (Fig. S6†).

We employed wide-angle XRD to characterize the composition and crystallinity of the obtained mesoporous nanocages. The intense peaks of the mesoporous nanocages centered at 39.8, 46.3, 67.8, 81.5 and 86.1° can be indexed to the (111), (200), (220), (311) and (222) planes of face-centered cubic (fcc) Pd, respectively (Fig. S7†). As the potential compounds of Bi in the reaction condition, the XRD profiles were firstly compared to the standard diffraction patterns of Bi, BiOCl and Bi_2_O_3_ crystals. No corresponding peaks are observed, ruling out residue or formation of the above-mentioned Bi-based crystals in the mesoporous nanocages. The shoulder peak observed at the left of the most intense peak is attributed to the expansion of the Pd crystal, which is probably induced by the incorporation of larger Bi atoms. These results indicate that both pure Pd and PdBi coexist in the mesoporous nanocages. For simplicity, we denote the sample as mesoporous PdBi nanocages (abbreviated as meso-PdBi nanocages). To identify the exact content and the distribution of the two components, the energy-dispersive X-ray spectrum (EDS) was recorded. The elemental mapping analysis shows that Pd and Bi are evenly distributed throughout the entire nanocages (Fig. 2d and e). The atomic ratio between Pd and Bi is confirmed to be 99.4 : 0.6, in agreement with the inductively coupled plasma-atomic emission spectrometry (ICP-AES) result. Due to the large difference in atomic content, only limited Pd lattices are penetrated by the Bi element, in good accordance with the intense Pd diffraction peaks and weak PdBi diffraction peak in XRD profiles.

**Fig. 3:**
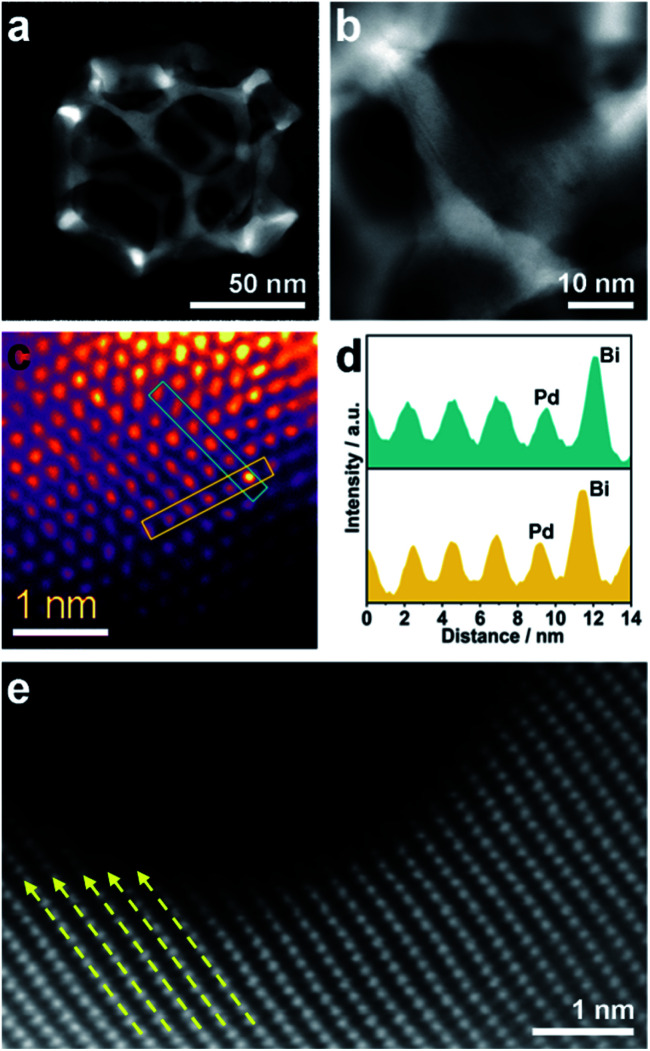
(a and b) HAADF-STEM images of an individual mesoporous PdBi nanocage. (c) High-resolution HAADF image of a surface region of the meso-PdBi nanocages. False color is applied to enhance the contrast. (d) Lattice profiles of the selected lines in panel (c). (e) Aberration-corrected HAADF image of the PdBi nanocage surface. The dashed arrows show the lattice distortion in panel (e).

Although the pore size and distribution are uniform, the nanoridges are randomly interconnected at vertices without an obvious orientation (Fig. 3a). The pore skeletons are twisted and curved, and different crystal planes are recognized around one individual pore (Fig. 3b). Due to the navigation effect of both the initially formed BiOCl spherical cores and spherical micelles, the formed PdBi ligaments are supposed to inherit the curvature of these two templates. To determine the structural effects of templates on the resultant PdBi ligaments, different sites were arbitrarily selected, and then the surface structure was further investigated using an aberration-corrected atomic-resolution scanning transmission electron microscope (STEM). The PdBi skeletons possess plenty of grain boundaries (Fig. S8a†) and the skeleton surface is not atomically smooth but consists of abundant kink and step sites (Fig. S8b†). Strain maps in several regions were further analyzed by geometrical phase analysis (GPA). It is very interesting that the more intense strains of meso-PdBi nanocages relative to meso-Pd NPs (without Bi doping) are directly proportional to the surface atomic defects. Detailed analysis of the strains reveals that tensile strain is the major type of strain (Fig. S9†), due to the incorporation of larger Bi atoms into the Pd lattices. Furthermore, Bi is present in the mesoporous PdBi nanocages as isolated Bi atoms, as revealed by the larger atomic size and brighter contrast (Fig. 3c and d). All the aforementioned surface structure and atomic arrangements result in obvious lattice distortion in the pore skeletons, which is probably caused by the concave porous structure and the penetration of larger Bi atoms into the Pd lattices (Fig. 3e). The resulting unique atomic structures have two positive effects on electrocatalytic properties; (i) the under-coordinated surface Pd kink and step sites^27,28^ and (ii) the strain effects integrating synergistic and ligand effects between Pd and Bi atoms. The incorporation of Bi atoms can alter the distance between the atomic arrangements of surface Pd atoms, usually favoring stronger adsorption of reaction intermediates.^29,30^ Therefore, our meso-PdBi nanocages are likely to improve electrocatalytic activity and durability by integrating a unique atomic structure with the mesoporous nanocage constructions.

**Fig. 4:**
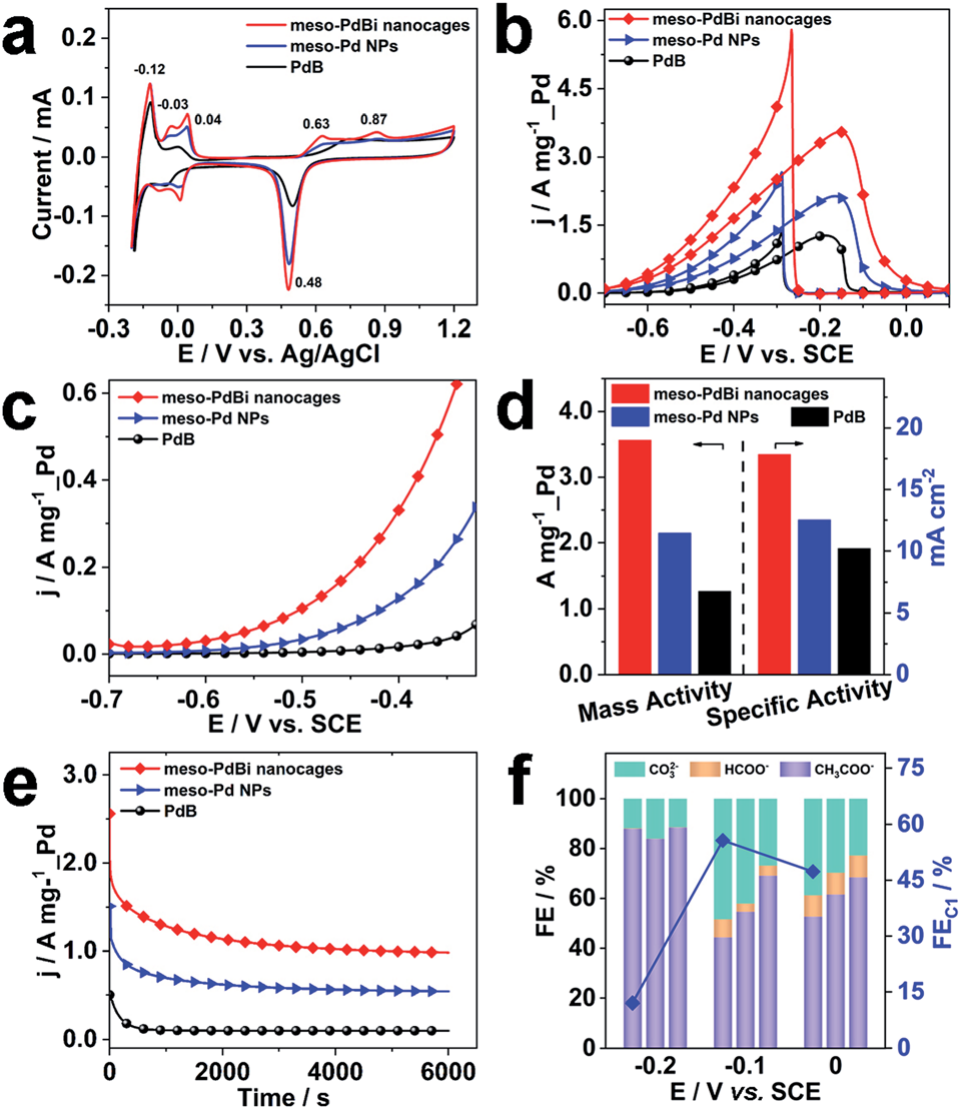
(a) CV curves recorded in 0.5 M H_2_SO_4_ at a scan rate of 50 mV s^−1^ of different electrocatalysts. (b) CV, (c) LSV and (e) chronoamperometric curves (recorded at −0.2 V) for the EOR in 1.0 M KOH containing 1.0 M C_2_H_5_OH. The CV and LSV curves were obtained at scan rates of 50 mV s^−1^ and 1.0 mV s^−1^, respectively. (d) Histogram of the mass and specific activities of different catalysts for the EOR. (f) FEs of the major products of the EOR at different potentials catalyzed by: meso-PdBi nanocages, meso-Pd NPs and PdB (from left to right for each potential).

Inspired by the highly open structures of meso-PdBi nanocages, the electrochemical surface area (ECSA) was determined to confirm the accessibility of the Pd active surfaces. For comparison, mesoporous Pd nanoparticles (abbreviated as meso-Pd NPs) (Fig. S10†) and commercial Pd black (PdB) were also studied under the same conditions. The ECSA values were estimated by calculating the charge for oxide reduction in cyclic voltammetry (CV) curves (Fig. 4a) assuming that the conversion factor for an oxide monolayer reduction is 420 μC cm^−2^.^31,32^ The obtained ECSA values are 33.28, 28.63 and 20.74 m^2^ g^−1^ for meso-PdBi nanocages, meso-Pd NPs and PdB samples, respectively. Moreover, the adsorption/desorption of HSO_4_^−^/SO_4_^2−^ and the oxide film formation behaviors are sensitive to the atomic arrangements of Pd surfaces. Thus, they are effective indicators of the surface atomic structures of the electrocatalysts. Both meso-PdBi nanocages and meso-Pd NPs exhibit CV profiles that are similar to those of the NPs with kink and step atomic sites,^33,34^ totally different from that of PdB. To be more specific, the apparent anodic peaks at 0.04 V and 0.87 V recognized in the CV profiles of meso-PdBi nanocages and meso-Pd NPs can be ascribed to the characteristics of Pd(311) surfaces with two atomic rows of the (100) terrace (Fig. 4a).^33,34^ The more intense peaks of meso-PdBi nanocages are evidence of the larger amount of surface atomic defects in meso-PdBi nanocages, consistent with the TEM observations (Fig. 3e).

To test the catalytic properties of the meso-PdBi nanocages, the ethanol oxidation reaction (EOR) was used as the model reaction. The EOR performance of all catalysts was evaluated in 1.0 M KOH containing 1.0 M ethanol. The corresponding CV profiles for the EOR catalyzed by the three catalysts at a sweep rate of 50 mV s^−1^ are shown in Fig. 4b. The mass activity of meso-PdBi nanocages (3.56 A mg^−1^_Pd) exceeds that of meso-Pd NPs (2.15 A mg^−1^_Pd) and PdB (1.27 A mg^−1^_Pd) (Fig. 4b and d). Even for the specific activity (the activity normalized by the Pd ECSA), the meso-PdBi nanocages have a superior activity of up to 17.82 mA cm^−2^ compared to the 12.51 and 10.20 mA cm^−2^ of meso-Pd NPs and PdB, indicating the increased utilization efficiency of the Pd catalytic sites (Fig. 4d). The steady state electrocatalytic study conducted by linear sweep voltammetry (LSV) at a scan rate of 1.0 mV s^−1^ shows that the meso-PdBi nanocages have a more negative onset potential and higher anodic current response at each potential, indicating easier and faster ethanol oxidation (Fig. 4c). The long-term stability of the catalysts was evaluated by chronoamperometry (CA). The current density of meso-PdBi nanocages decays more slowly relative to that of meso-Pd NPs and PdB, suggesting that meso-PdBi nanocages have higher durability during electrochemical measurements (Fig. 4e). This can be supported by a perfect retention of the original structure and composition even after the stability measurement. As seen in Fig. S11,† no difference is observed in the SEM and EDS results before and after the electrocatalytic test.

Complete oxidation of ethanol to CO_2_*via* a 12-electron process by breaking the C–C bond endows the fuel cell technology with higher efficiency. Normally used Pd- or Pt-based electrocatalysts, however, are usually prone to form CH_3_COOH/CH_3_CHO *via* an incomplete C2 oxidation process.^35,36^ Therefore, the CO_2_ selectivity is an important indicator of the catalytic efficiency of electrocatalysts toward the EOR. To check the CO_2_ selectivity, the accumulated products of the EOR at potentials close to the peaks of the CV plots were quantitatively analyzed by ^1^H nuclear magnetic resonance (^1^H-NMR) and ion chromatography, respectively. The peaks with chemical shifts of *δ* = 1.95 and *δ* = 8.50 ppm in the ^1^H-NMR spectrum and the peaks with elution times of 13.50 and 15.38 min in ion chromatography are ascribed to CH_3_COO^−^ and HCOO^−^, respectively (Fig. S12 and S13†). The faradaic efficiency (FE) of CO_2_ was calculated by deducting the FEs of formate and acetate products because its reaction with KOH electrolyte and CO_2_ contamination from the atmosphere limit the accurate direct detection of CO_2_. Particularly, the FEs of the major EOR products catalyzed by the meso-PdBi nanocages, meso-Pd NPs and PdB at the indicated potentials (−0.2 V, −0.1 V and 0.0 V) are shown and compared (Fig. 4f). When PdB is used as a catalyst, CH_3_COO^−^ is the dominant product, demonstrating that PdB preferentially catalyzes *via* the C2 pathway. In contrast, both meso-PdBi nanocages and meso-Pd NPs suppress the formation of C2 products at all the studied potentials. The FEs for C1 products are 12.06, 55.69 and 47.33 at −0.2, −0.1 and 0.0 V over meso-PdBi nanocages, exceeding that of meso-Pd NPs (16.16, 45.40 and 38.46) and PdB (11.58, 30.91 and 31.57). The abundant Pd kink/step sites on the concave surface of meso-PdBi nanocages are thought to play a major role in the cleavage of the C–C bond to form the adsorbed CO and other fragments because the EOR is a structure sensitive reaction. The adsorbed reaction intermediates are then completely oxidized to CO_2_ through a Langmuir–Hinshelwood process with the assistance of adsorbed OH radicals on the aerobic Bi sites.^37,38^

**Fig. 5:**
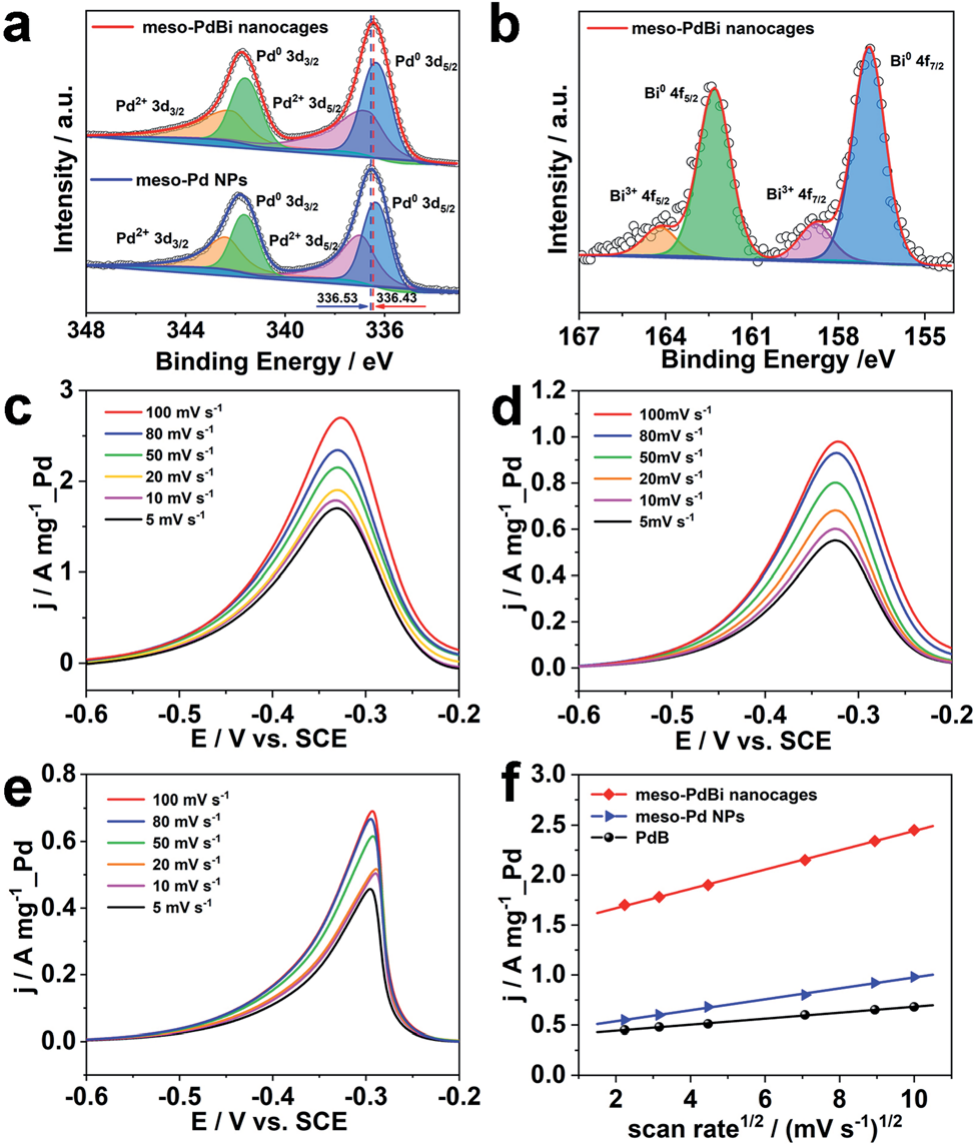
XPS patterns of (a) Pd 3d and (b) Bi 4f of meso-PdBi nanocages and meso-Pd NPs. (c–e) LSVs of the EOR catalyzed by (c) meso-PdBi nanocages, (d) meso-Pd and (e) PdB in 1.0 M KOH containing 1.0 M C_2_H_5_OH at different scan rates. (f) The relationship of the peak current *versus* the square root of the scan rate.

Further studies were performed to reveal the intrinsic mechanism responsible for the superior electrocatalytic performance of the meso-PdBi nanocages. X-ray photoelectron spectroscopy (XPS) measurements were carried out to obtain detailed information about the electronic states of different elements. The surface atomic ratio of Pd to Bi obtained from XPS is 99.3 : 0.7, slightly higher than that of the overall PdBi composition (99.4 : 0.6), revealing that the Bi preferentially distributes on the surface of the mesoporous skeletons. The binding energies of Pd 3d peaks negatively shift 0.2 eV in the meso-PdBi nanocages relative to that in meso-Pd NPs (Fig. 5a), which is due to the electron donation effect of the Bi element with less electronegativity. The Bi 4f peaks also show a right shift in comparison to the values reported (Fig. 5b).^39,40^ The shift in the binding energies of Pd and Bi elements observed in meso-PdBi nanocages is ascribed to the unique Pd–Bi local bonding unit caused by the strong charge exchange between Pd-4d and Bi-(sp) orbitals.^41^ To specifically distinguish the electronic structures, the Pd 3d XPS peaks were deconvolved to four peaks (Fig. 5a) based on the constraint that the doublet peak components in the 3d_5/2_ and 3d_3/2_ regions have a separation of 5.26 eV and area ratio of 3/2. From the XPS result, we conclude that a strong electron change is observed in the meso-PdBi nanocages, probably evoking both better electrocatalytic activity and durability. The mass transfer features of the EOR catalyzed by different Pd-based materials were explored using CV methods. The forward peak current densities of the EOR increase with increasing scan rates (Fig. 5c–e). The peak current density has a linear dependence on the square root of scan rates, suggesting that the EOR catalyzed by all the materials follows a diffusion-controlled process.^42,43^ In this case, the slope can directly reveal the diffusion efficiency. The higher slope value of meso-PdBi nanocages in comparison to meso-Pd NPs and PdB demonstrates the improved electron and mass transfer of the meso-PdBi nanocages (Fig. 5f and Table S1†).^43^

## Conclusion

We have demonstrated a novel and expedient strategy for nanoarchitecting mesoporous PdBi nanocages by precisely controlling Bi^3+^ hydrolysis to generate precipitate cores *in situ* as templates for the growth of mesoporous shells. The mesoporous nanocages composed of thin nanoridges can be accessed from all directions rather than just the outer surface. Thanks to the concave surfaces of the mesoporous skeletons and the alloying of Bi with Pd lattices, both kink/step sites and lattice strains are found on the surfaces of meso-PdBi nanocages. By virtue of the synergistic effects of Pd and Bi atoms within the same crystal on the adsorption of reaction intermediates, the mesoporous nanocages have an improved electrocatalytic performance toward ethanol oxidation with relatively high activity, superior durability and enhanced complete oxidation of ethanol *via* the C1 pathway (Table S2†). We believe this approach is a facile and versatile method for creating highly open nanostructures with controlled rational structures and compositions for targeted electrocatalytic applications by integrating the micelle assembly approach with the hydrolysis effect of metal ions.

## Experimental section

### Chemicals

Poly(styrene)-*b*-poly(ethylene oxide) (PS_5000_-*b*-PEO_2200_) was purchased from Polymer Source, Inc. Palladium(ii) chloride (PdCl_2_, 99.999%) was obtained from Alfa Aesar. Pd black and Nafion solution (5% in a mixture of lower aliphatic alcohols and water) were purchased from Sigma Aldrich. Bismuth chloride (BiCl_3_) and tetrahydrofuran (THF) were purchased from Aladdin Industrial Corporation. Hydrazine hydrate (80%) Tianjin Komil Chemical Reagent Co., Ltd. Hydrochloric acid (HCl, 36–38%) was purchased from Tianjin Fuyu Fine Chemicals Co. Ltd. Before experiments, 0.1 g of deep brown PdCl_2_ powder was dissolved in 0.46 mL concentrated HCl solution and 6.58 mL ultrapure water to obtain an 80 mM H_2_PdCl_4_ solution. H_3_BiCl_6_ solution was prepared by dissolving 0.1 g of white BiCl_3_ powder in 0.34 mL concentrated HCl solution and 3.62 mL ultrapure water. Ultrapure water (Milli-Q Integral 5, 18.0 MΩ cm resistivity) was used in all experiments. All chemicals were used without further purification.

### Preparation of mesoporous PdBi nanoparticles (meso-PdBi NPs)

Meso-PdBi NPs were synthesized *via* a wet chemical reduction process. In a typical synthesis, 4 mg of PS_5000_-*b*-PEO_2200_ was completely dissolved in 0.2 mL THF. Next, 0.95 mL DI water was added to stimulate the micellization of block copolymers, after which 0.1 mL HCl (6 M), 0.225 mL H_2_PdCl_4_ (80 mM), 0.025 mL BiCl_3_ solution (80 mM) and 0.5 mL hydrazine hydrate (0.2 M) were added in sequence. Finally, the reaction solution was incubated in a water bath for 10 h at 60 °C. The obtained black product was collected by centrifugation at 14 000 rpm for 10 min and the block copolymer was removed by repeatedly washing/centrifugation with THF and ethanol.

### Preparation of mesoporous PdBi nanocages (meso-PdBi nanocages)

Meso-PdBi nanocages were prepared by acid etching treatment. The obtained meso-PdBi NPs were immersed in 10 M HCl aqueous solution for 12 h to completely dissolve the BiOCl cores. Finally, the precipitates were thoroughly washed with ultrapure water.

### Preparation of mesoporous Pd nanoparticles (meso-Pd NPs)

The meso-Pd NPs were synthesized under the same conditions as mesoporous PdBi nanoparticles without using BiCl_3_.

### Characterization

Scanning electron microscope (SEM) images were obtained using a Field emission scanning electron microscope (Zeiss Supra55) with an accelerating voltage of 10 kV. Transmission electron microscopy (TEM) and high-angle annular dark-field scanning TEM (HAADF-STEM) images were taken using a Talos F200X G2 microscope at an accelerating voltage of 200 kV. The atomically resolved HAADF results were obtained using the STEM mode of a FEI Themis Z with double aberration correctors. Wide-angle powder X-ray diffraction (XRD) profiles were recorded with a Bruker D8 Advance diffractometer with Cu-Kα radiation (*λ* = 1.54178 Å). X-ray photoelectron spectroscopy (XPS) was performed using a PHI 5000 VersaProbe III with a monochromatic Al Kα X-ray source with the beam size of 200 μm. Charge compensation was achieved by dual beam charge neutralization and the binding energy was corrected by setting the binding energy of the hydrocarbon C 1s feature to 284.8 eV.

### Electrochemical analysis

Electrochemical experiments were performed with a CHI760E workstation. A conventional three-electrode cell was used with a saturated calomel electrode (SCE), Pt wire and glassy carbon electrode (GCE) as the reference electrode, counter electrode and working electrode, respectively. Prior to surface coating, the GCE was polished with 1.0 and 0.05 μm alumina powder, rinsed with ultrapure water and dried under a nitrogen gas flow. The working electrode was prepared by dropping 3 μL of catalyst suspension (1 mg mL^−1^) onto the surface of the GCE with 3 mm diameter. After drying under atmospheric conditions, a Nafion solution (3.0 μL, 0.05 wt%) was subsequently coated on the surface and dried at ambient temperature. The ethanol oxidation reaction was carried out in 1.0 M KOH solution containing 1.0 M ethanol. The electrochemical surface area (ECSA) was determined using cyclic voltammograms (CVs) between −0.2 V and 1.2 V (*vs.* Ag/AgCl) in 0.5 M H_2_SO_4_. The oxide reduction charge was used to calculate the ECSA based on an assumption that the conversion factor for an oxide monolayer reduction is 420 μC cm^−2^ on a smooth Pd surface.

### Product detection

The experimental setup for checking C1 selectivity was operated in a gastight cell, in which N_2_ was delivered continuously to the electrolyte with a flow rate of 20 sccm. With the aid of the N_2_ flow, the gaseous products were directly injected into a gas chromatograph (GC) at each 30 min for gas product identification and quantification. For quantifying the liquid products, the electrolyte after chronoamperometric measurements at indicated potentials for 1.0 h was collected and analyzed by ^1^H nuclear magnetic resonance (^1^H-NMR) and Ion Chromatography, respectively. For ^1^H-NMR analysis, 0.5 mL of electrolyte was mixed with 0.1 mL of D_2_O (DMSO concentration of 100 ppm). The Ion Chromatograph was equipped with a 930 Compact IC flex and 919 autosampler plus. Separations were performed on a Metrosep organic Acid-250/7.8 chromatographic column, coupled with a Metrosep RP2 Guard column. The 5 mM H_2_SO_4_ and 100 mM LiCl solutions were used as the eluent and regenerant, respectively. An intelligent conductivity detector with a chemical suppressor (MSM) was used for detection. Calibration standards (formic acid and acetic acid) were prepared in a series of concentrations, and the linear correlation coefficient was >99.9%. In the case of ion chromatography detection, 5.0 mL of electrolyte was diluted with 5.0 mL H_2_O. After the quantification, the faradaic efficiencies (FEs) of the products were calculated as follows:
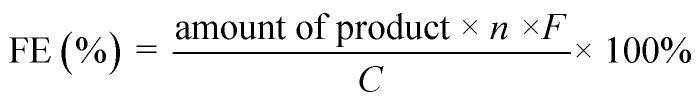
where *n* is the number of moles of electrons to participate in the faradaic reaction, *F* is the Faraday constant (96 485 C mol^−1^), and *C* is the amount of charge passed through the working electrode.

## Data availability

All relevant data are presented in the main text and ESI.†

## Author contributions

D. D. performed most of the experiments. Q. G. draw the scheme. L. M., S. R., J.-X. L., Q. H. and L. F. carried out the EOR measurement and calculated the C1 selectivity. W. D. and R. S. carried out TEM measurements and strain analysis. X. W., C. L. and Y. Y. discussed the results. C. L. wrote the manuscript.

## Conflicts of interest

There are no conflicts to declare.

## Supplementary Material

SC-013-D1SC06314F-s001
