# Supplementary material for: Mesoporous PdBi nanocages for enhanced electrocatalytic performances by all-direction accessibility and steric site activation
Source: Chem Sci. 2022 Feb 28;13(13):3819–25. doi: 10.1039/d1sc06314f (PMC8966753; doi:10.1039/d1sc06314f)
Supplement: SC-013-D1SC06314F-s001 [file SC-013-D1SC06314F-s001.pdf]

Electronic Supplementary Material (ESI) for Chemical Science.
This journal is © The Royal Society of Chemistry 2022

Mesoporous PdBi Nanocages for Enhanced Electrocatalytic Performances by All-Direction Accessibility and Steric Site Activation

Dawei Du,[a] Qinghong Geng,[a] Lian Ma,[a] Siyu Ren,[a] Jun-Xuan Li,[a] Weikang Dong,[b] Qingfeng Hua,[a] Longlong Fan,[a] Ruiwen Shao,[b] Xiaoming Wang,[c] Cuiling Li,*[a] and Yusuke Yamauchi,*[d,e]

[a] Key Laboratory of Cluster Science, Ministry of Education, Beijing Key Laboratory of Photoelectronic/Electrophotonic Conversion Materials, School of Chemistry and Chemical Engineering, Beijing Institute of Technology, Beijing 100081, China

[b] Beijing Advanced Innovation Center for Intelligent Robots and Systems and Institute of Engineering Medicine, Beijing Institute of Technology, Beijing 100081, China

[c] Key Laboratory for Preparation and Application of Ordered Structural Materials of Guangdong Province, Department of Chemistry, Shantou University, Shantou 515063, China

[d] International Center for Materials Nanoarchitectonics (MANA), National Institute for Materials Science (NIMS), Tsukuba 305-0044, Japan

E-mail: y.yamauchi@uq.edu.au

[e] School of Chemical Engineering and Australian Institute for Bioengineering and Nanotechnology (AIBN), The University of Queensland, Brisbane 4072, Australia

E-mail: licuiling@bit.edu.cn

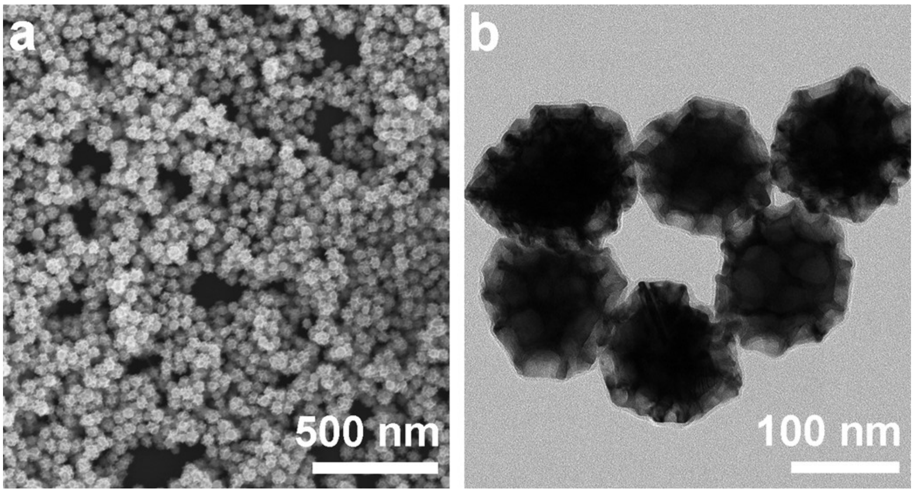

Figure S1 (a) SEM and (b) TEM images of the prepared nanoparticles before etching treatment.

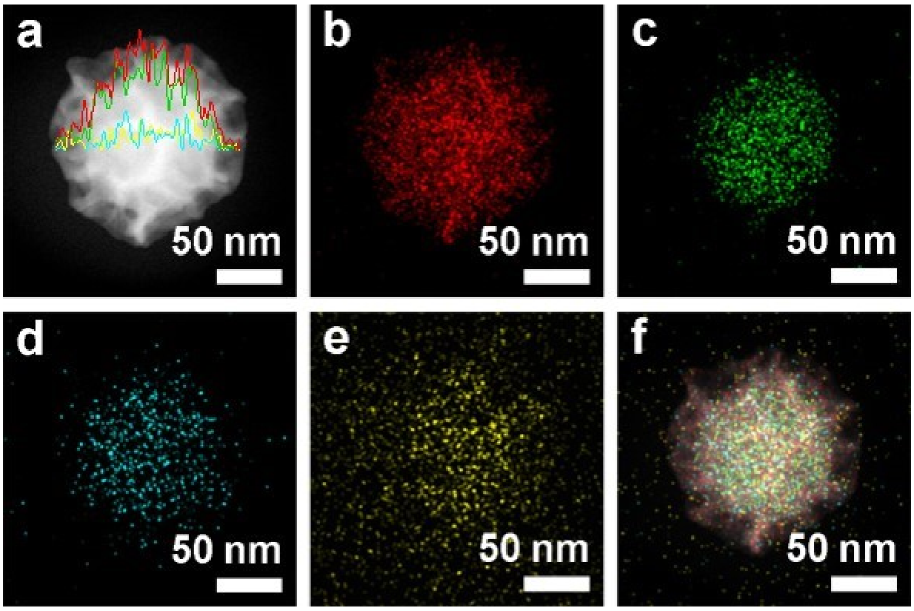

Figure S2 (a) HAADF-STEM image and corresponding elemental maps: (b) Pd, (c) Bi, (d) Cl, (e) O and (f) overlap. Line profiles of elements across the whole nanoparticle are shown in panel (a).

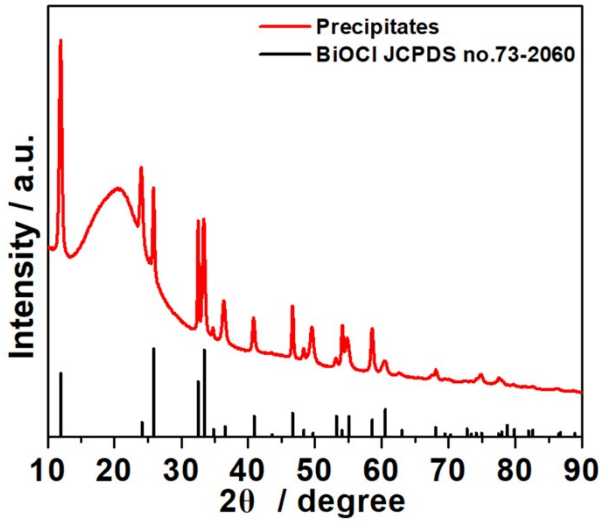

Figure S3 XRD patterns of the initial precipitates formed under the reaction condition.

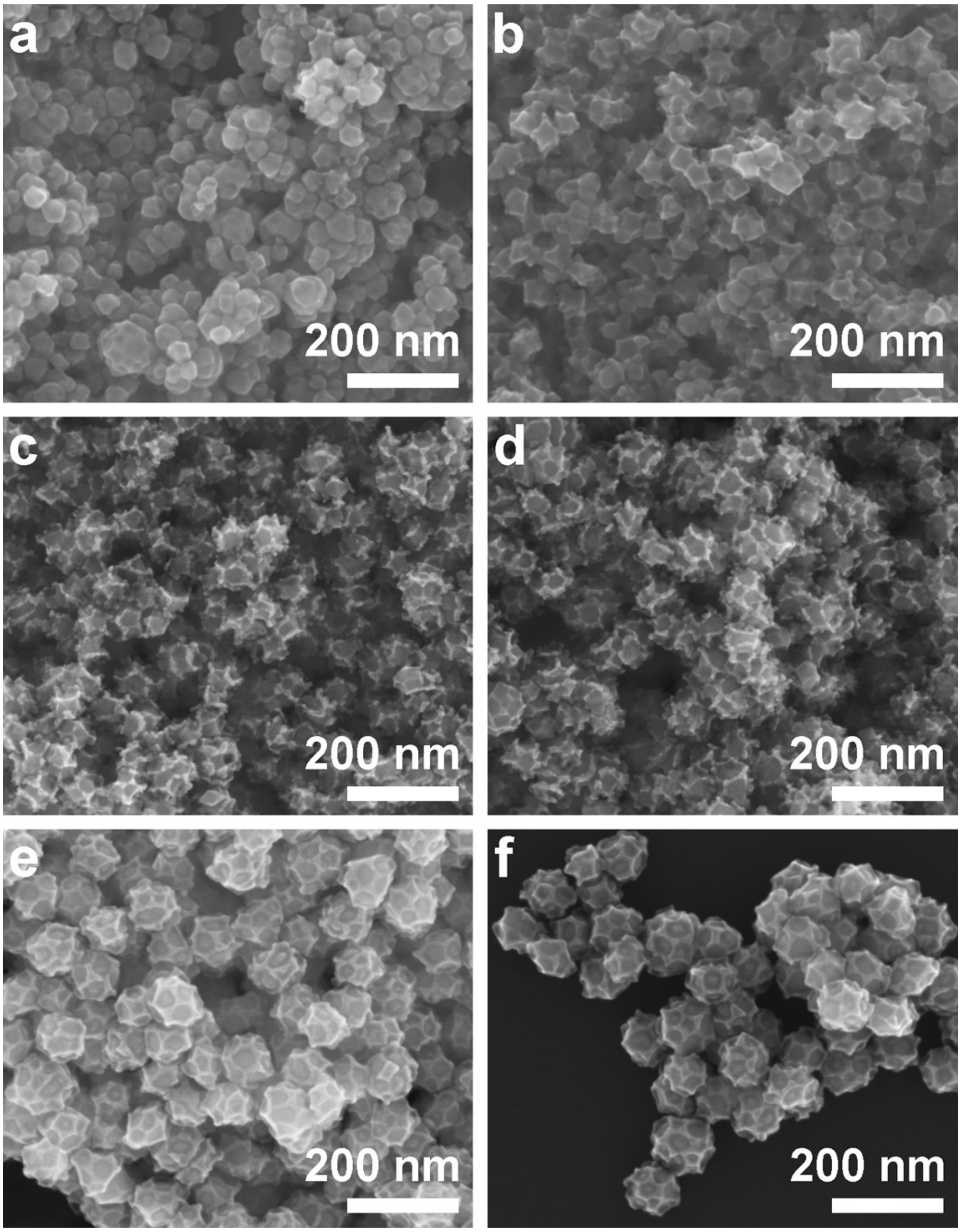

Figure S4 Typical SEM images of the products collected at different reaction times: (a) 2 min, (b) 3 min, (c) 5 min, (d) 8 min, (e) 15 min, and (f) 30 min.

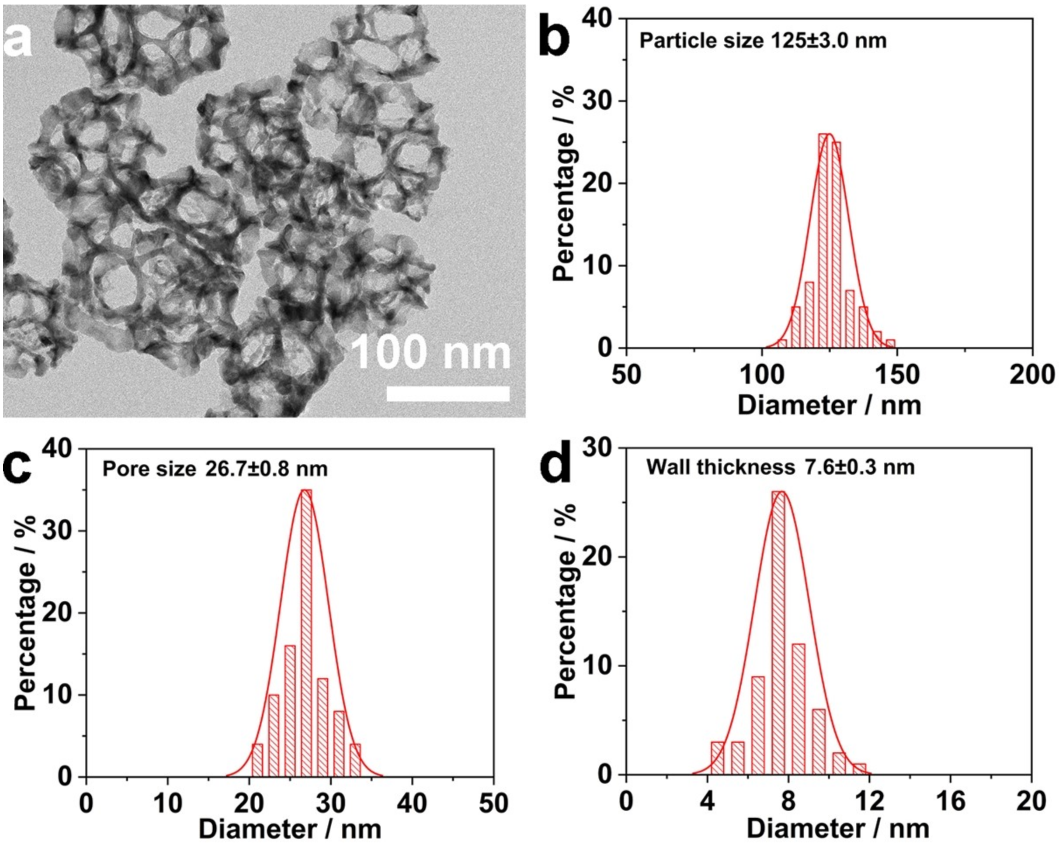

Figure S5 (a) TEM image of mesoporous nanocages and histograms of (b) particle size, (c) pore size and (d) wall thickness distributions.

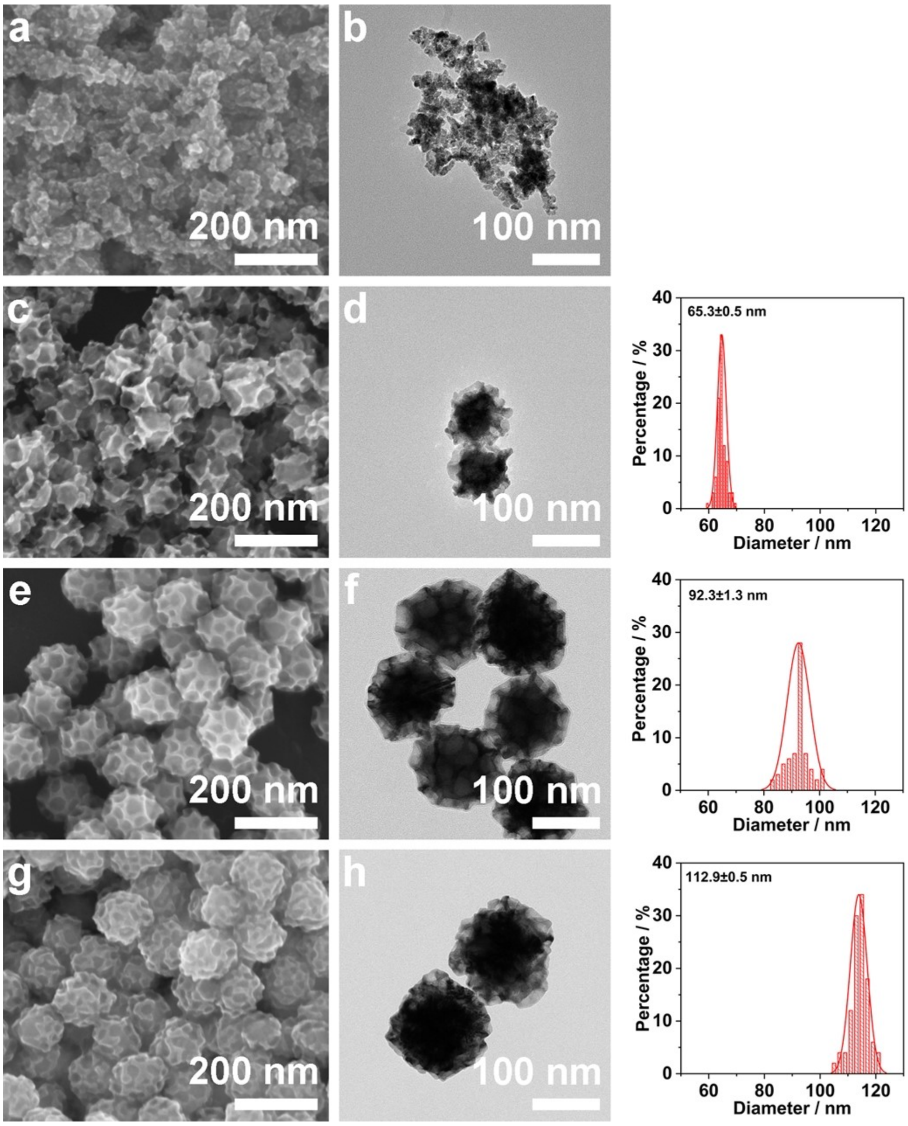

Figure S6 (a, c, e, g) SEM and (b, d, f, h) TEM images of the mesoporous nanoparticles after adding different amount of HCl: (a, b) 0 mL, (c, d) 0.05 mL, (e, f) 0.1 mL and (g, h) 0.2 mL, respectively. The histograms of core size distributions are displayed in the right column.

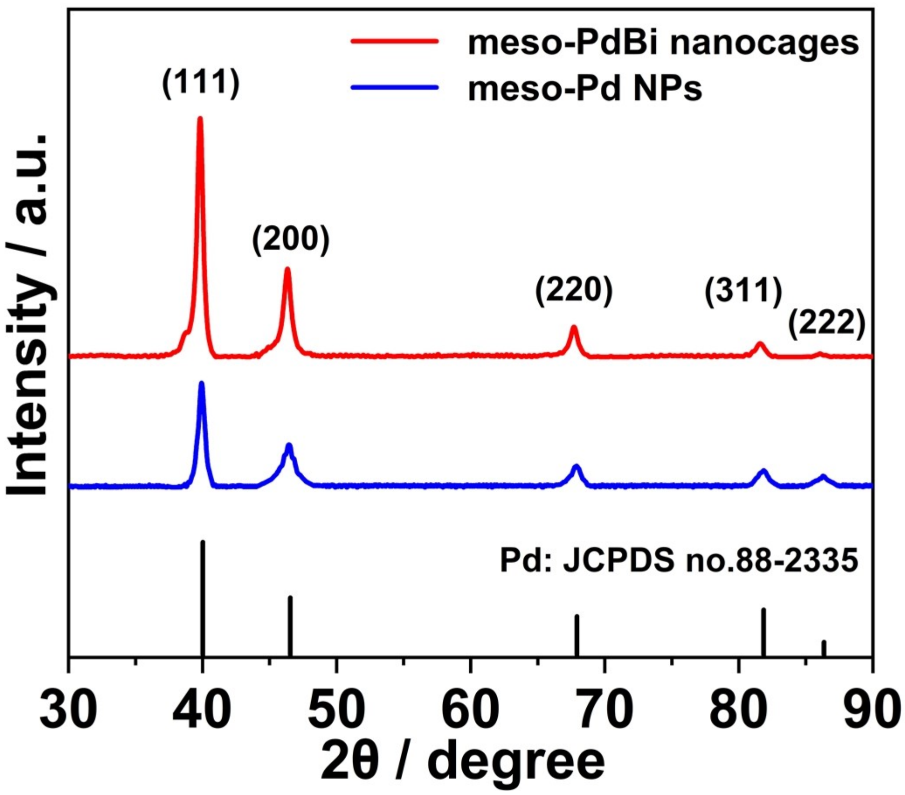

Figure S7 XRD patterns of meso-PdBi nanocages and meso-Pd NPs.

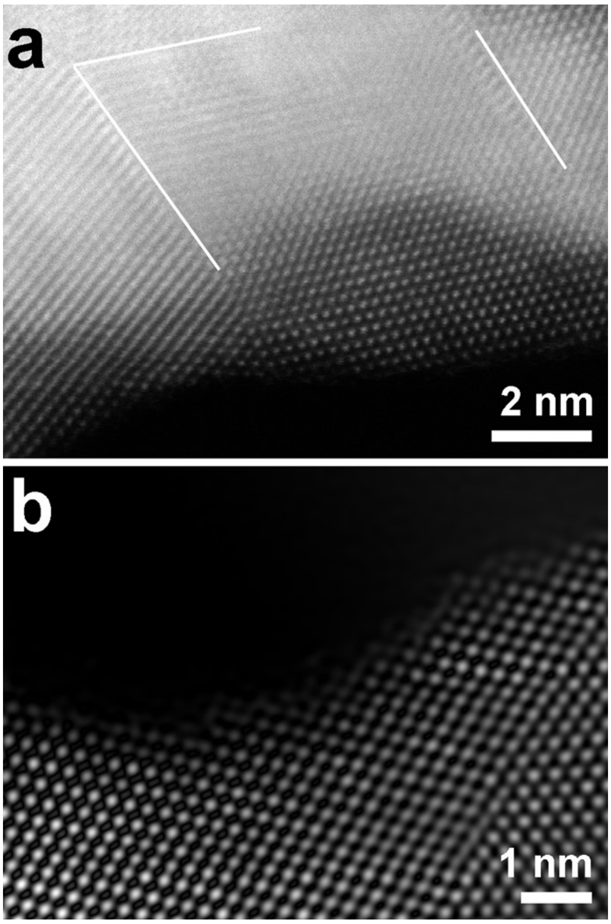

Figure S8 (a) Aberration-corrected HAADF-STEM images of different locations on the meso-PdBi nanocages. (b) A clear image on the concave curvature.

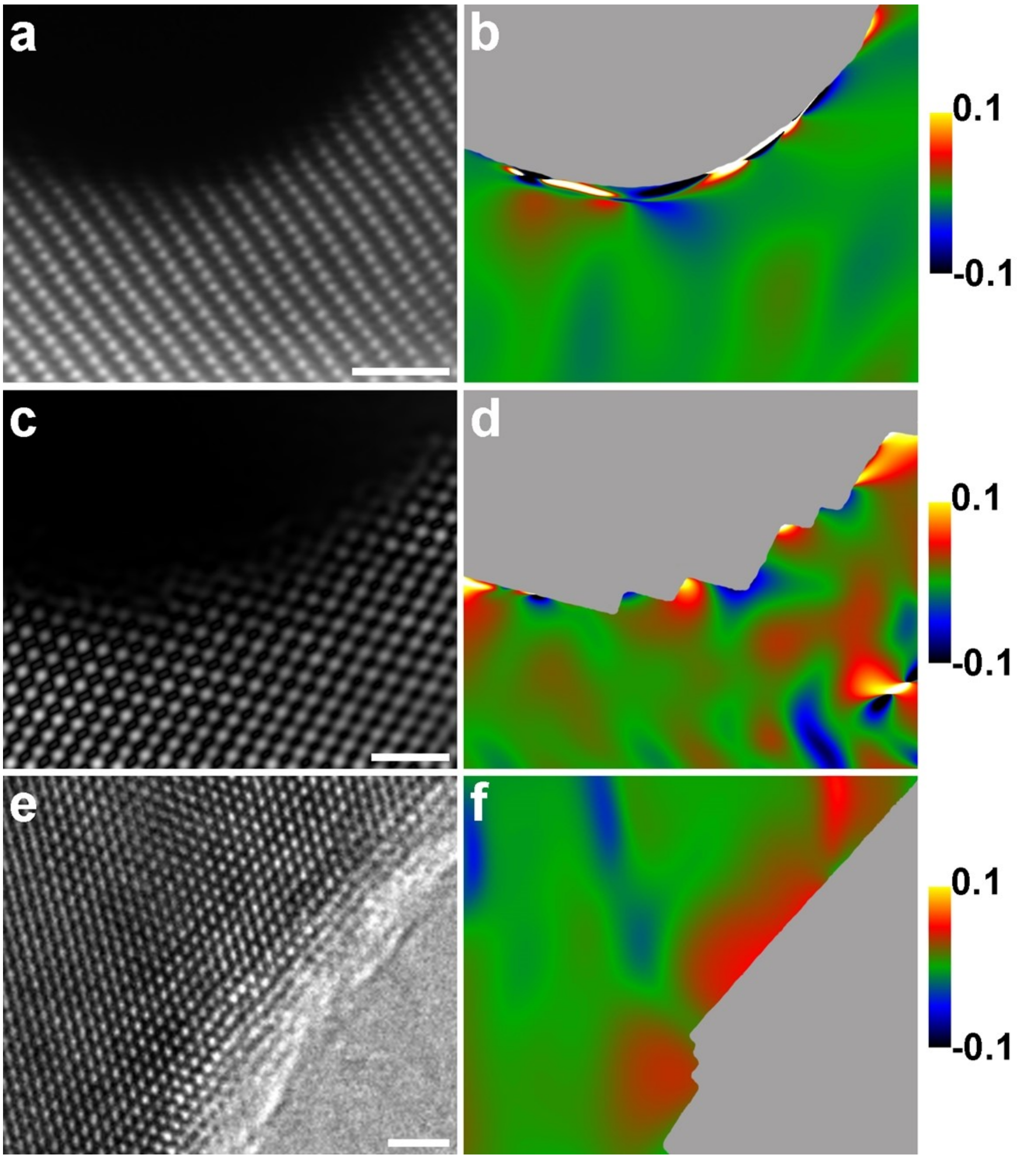

Figure S9 (a, c, e) Representative aberration-corrected HAADF images of surface structures of (a, c) meso-PdBi nanocages and (e) meso-Pd NPs, respectively. (b, d, f) Strain maps of ε_{xx} obtained by geometrical phase analysis (GPA) for (b, d) meso-PdBi nanocages and (f) meso-Pd NPs. Negative values represent compressive strain, while positive values depict tensile strain.

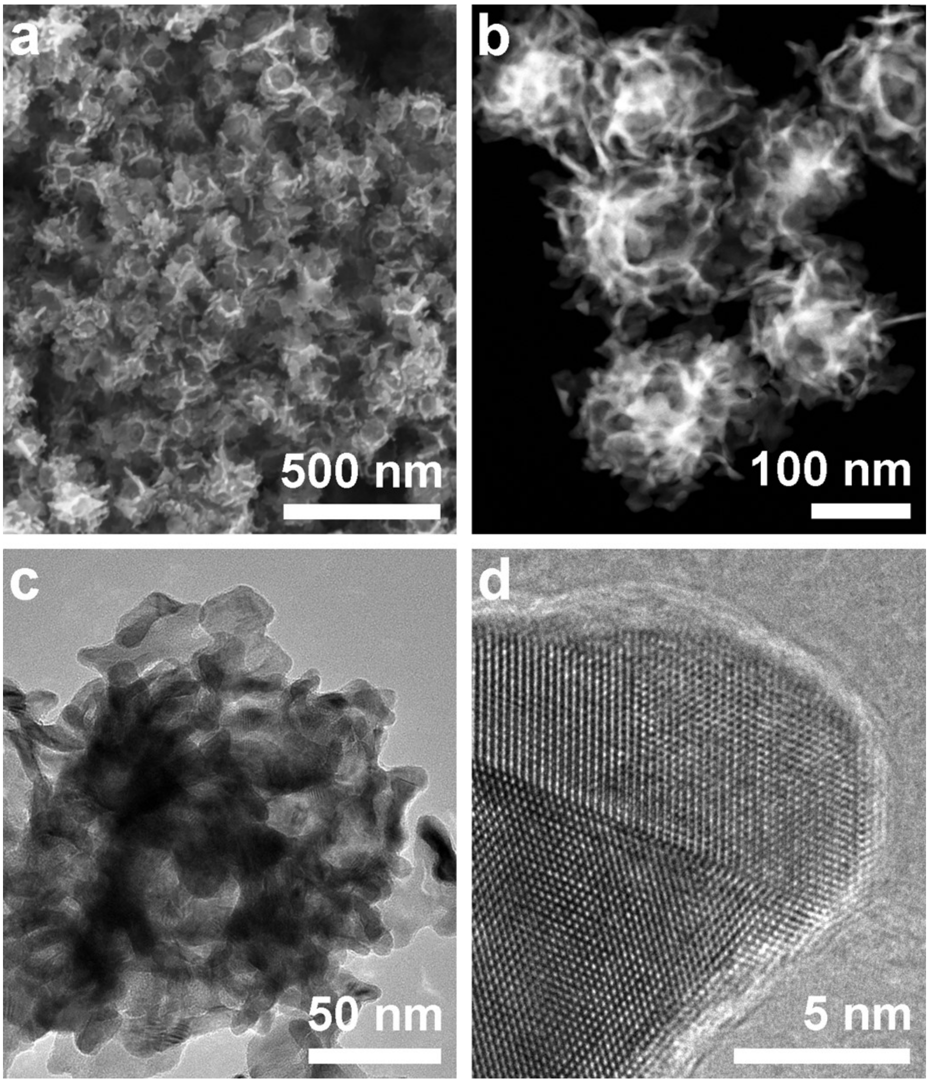

Figure S10 (a) SEM, (b) HAADF-STEM, (c) TEM and (d) HRTEM images of the meso-Pd NPs.

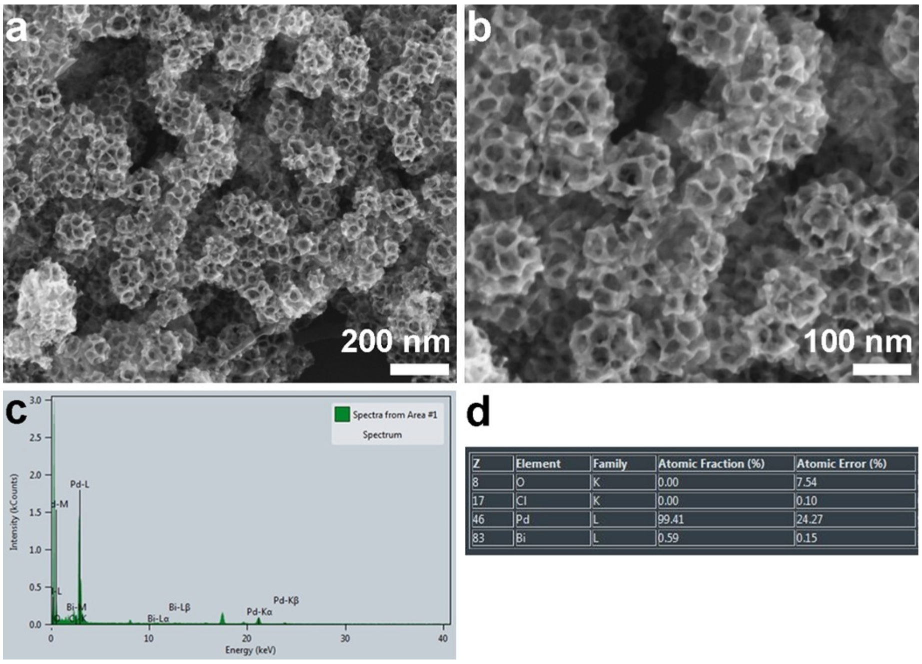

Z	Element	Family	Atomic Fraction (%)	Atomic Error (%)
8	O	K	0.00	7.54
17	Cl	K	0.00	0.10
46	Pd	L	99.41	24.27
83	Bi	L	0.59	0.15

Fig. S11 (a, b) SEM images and (c) EDS result of the meso-PdBi nanocages after *i-t* measurement in 1.0 M KOH containing 1.0 M C_2H_5OH. (d) The corresponding atomic ratios of different elements derived from EDS analysis.

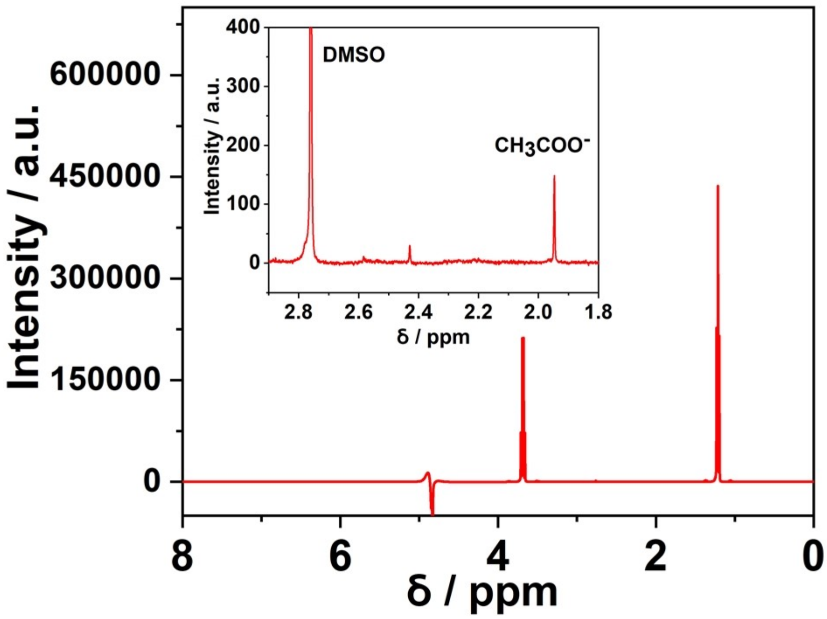

Figure S12 Representative ^{1}H-NMR spectra of the electrolyte after EOR catalyzed by meso-PdBi nanocages at -0.1 V *vs.* SCE for 1.0 h. DMSO is used as an internal standard for quantification of CH_3COO^- and $HCOO^-$.

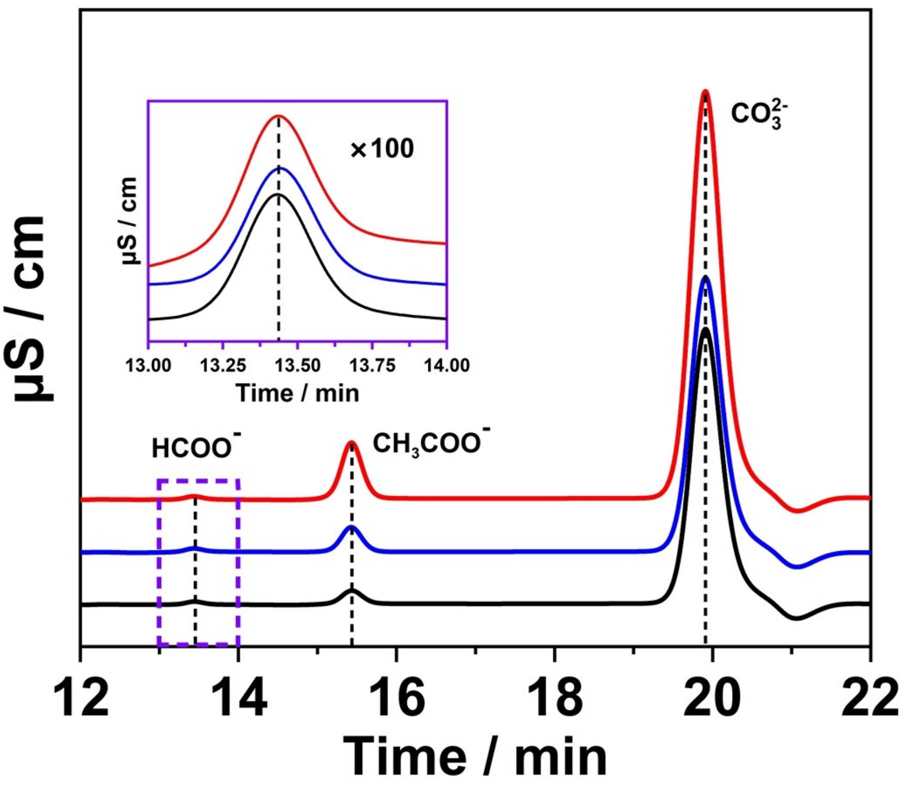

Figure S13 Representative ion chromatographic spectra of the electrolyte after EOR catalyzed by meso-PdBi nanocages (red), meso-Pd NPs (blue) and PdB (black) at -0.1 V *vs.* SCE for 1.0 h. The inset shows a magnification of $HCOO^-$ peaks.

Table S1 Comparison of EOR performance. The data was derived from **Fig. 5c-f**.

**Sample Name**	**Slope (mA/(V $s^{-1})^{1/2}$)**	**R^2**
meso-PdBi nanocages	96.77	0.999
meso-Pd NPs	54.80	0.998
PdB	29.89	0.998

Table S2 Comparison of EOR performances of meso-PdBi nanocages with previously reported catalysts.

Sample name	Electrolyte	Scan rate (mV s^{-1})	Mass activity (A mg^{-1}_Pd)	Specific activity (mA cm^{-2})	C1 Selectivity	References
Meso-PdBi nanocages	1.0M KOH + 1.0 M C_2H_5OH	50	3.56	17.82	55.7%	This work
PdCo nanotube array	1.0M NaOH + 1.0 M C_2H_5OH	50	1.49	--	--	1
Pd/TiO_2 nanotube array	2.0M KOH + 10 wt% C_2H_5OH	50	--	14.1	--	2
PdNiP ternary NPs	1.0M NOH + 1.0 M C_2H_5OH	100	4.95	--	--	3
Tetrahexahedra l Pd NCs	0.1M NaOH + 0.1 M C_2H_5OH	10	--	3.83	--	4
Pd icosahedra	0.1M NaOH + 1.0 M C_2H_5OH	50	--	0.8	--	5
Pd/C	1.0M NaOH + 1.0 M C_2H_5OH	50	--	16	--	6
Ordered Pd_2Ge intermetallic NPs	1.0M KOH + 1.0 M C_2H_5OH	50	--	4.1	--	7
Ternary PdBP mesoporous NSs	1.0M KOH + 1.0 M C_2H_5OH	50	3.65	--	--	8
PdBi single-atom alloy aerogels	1.0M KOH + 1.0 M C_2H_5OH	50	5.5	--	0.0%	9
SnO_2-Rh nanosheets	0.1M KOH + 0.5 M C_2H_5OH	50	--	--	72.8%	10
Ag@$Pd_2P_{0.2}$	1.0M KOH + 1.0 M C_2H_5OH	50	7.24	6.68	19.0%	11

References

[1] A.-L. Wang, X.-J. He, X.-F. Lu, H. Xu, Y.-X. Tong, G.-R. Li, *Angew. Chem. Int. Ed.* **2015**, *54*, 3669-3676.

[2] Y.-X. Chen, A. Lavacchi, S.-P. Chen, F. Benedetto, M. Bevilacqua, C. Bianchini, P. Fornasiero, M. Innocenti, M. Marelli, W. Oberhauser, S.-G. Sun, F. Vizza, *Angew. Chem. Int. Ed.* **2012**, *51*, 8500-8504.

[3] L. Chen, L. Lu, H. Zhu, Y. Chen, Y. Huang, Y. Li, L. Wang, *Nat. Commun.* **2017**, *8*, 14136.

[4] N. Tian, Z.-Y. Zhou, N.-F. Yu, L.-Y. Wang, S.-G. Sun, *J. Am. Chem. Soc.* **2010**, *132*, 7580-7581.

[5] G. Fu, X. Jiang, L. Tao, Y. Chen, J. Lin, Y. Zhou, Y. Tang, T. Lu, T. *Langmuir* **2013**, *29*, 4413-4420.

[6] G. Cui, S. Song, P. Shen, A. Kowal, C. Bianchini, *J. Phys. Chem. C* **2009**, *113*, 15639-15642.

[7] S. Sarkar, R. Jana, Suchitra, U. V. Waghmare, B. Kuppan, S. Sampath, S. C. Peter, *Chem. Mater.* **2015**, *27*, 7459-7476.

[8] H. Lv, D. Xu, L. Sun, J. Henzie, S. L. Suib, Y. Yamauchi, B. Liu, *ACS Nano* **2019**, *13*, 12052-12061.

[9] H. Wang, L. Jiao, L. Zheng, Q. Fang, Y. Qin, X. Luo, X. Wei, L. Hu, W. Gu, J. Wen, C. Zhu, *Adv. Funct. Mater.* **2021**, 2103465.

[10] S. Bai, Y. Xu, K. Cao, X. Huang, Adv. Mater. **2020**, 2005767.

[11] X. Yang, Z. Liang, S. Chen, M. Ma, Q. Wang, X. Tong, Q. Zhang, J. Ye, L. Gu, N. Yang, N. *Small* **2020**, *16*, 2004727.
